# Identification of Target Body Composition Parameters by Dual-Energy X-Ray Absorptiometry, Bioelectrical Impedance, and Ultrasonography to Detect Older Adults With Frailty and Prefrailty Status Using a Mobile App in Primary Care Services: Descriptive Cross-Sectional Study

**DOI:** 10.2196/67982

**Published:** 2025-05-15

**Authors:** Beatriz Ortiz-Navarro, José Losa-Reyna, Veronica Mihaiescu-Ion, Jerónimo Garcia-Romero, Margarita Carrillo de Albornoz-Gil, Alejandro Galán-Mercant

**Affiliations:** 1 Public Andalusian Health System, Málaga, Spain; 2 CIBER of Frailty and Healthy Aging, Madrid, Spain; 3 Sports Science Research Centre, King Juan Carlos University, Madrid, Spain; 4 Institute of Biomedicine of Cádiz, Cádiz, Spain; 5 MOVE-IT Research Group, Department of Nursing and Physiotherapy, Faculty of Health Sciences, University of Cádiz, Cádiz, Spain; 6 Área de Educación Física y Deportiva, Facultad de Medicina, Universidad de Málaga, Boulevard Louis Pasteur, Málaga, 29071, Spain, 34 952131574

**Keywords:** frailty syndrome, older adults, body composition, bioelectrical impedance analysis, muscle ultrasound, dual-energy X-Ray absorptiometry, mobile health apps, primary care, PowerFrail App

## Abstract

**Background:**

Frailty syndrome in older adults represents a significant public health concern, characterized by a reduction in physiological reserves and an increased susceptibility to stressors. This can result in adverse health outcomes, including falls, hospitalization, disability, and mortality. The early identification and management of frailty are essential for improving quality of life and reducing health care costs. Conventional assessment techniques, including dual-energy X-ray absorptiometry (DXA), bioelectrical impedance analysis (BIA), and muscle ultrasound (US), are efficacious but frequently constrained in primary care settings by financial and accessibility limitations.

**Objective:**

The aim of this study is to analyze the differences in anthropometric characteristics, physical function, nutritional status, cognitive status, and body composition among older adults identified as frail, prefrail, or robust in primary care services using the PowerFrail mobile app. Furthermore, the study assesses the predictive capacity of body composition variables (whole-body phase angle [WBPhA] via BIA, US-measured rectus femoris muscle thickness, and DXA-derived lean mass) in identifying frailty and evaluates their feasibility for implementation in primary care.

**Methods:**

A descriptive cross-sectional study was conducted with 94 older adult participants aged between 70 and 80 years, recruited through the Andalusian Health Service in Spain. Frailty status was classified using the PowerFrail App, which integrates muscle power assessment and provides personalized physical activity recommendations. Body composition was measured using WBPhA (BIA), muscle US, and DXA. Statistical analyses included 1-way ANOVA for group comparisons, logistic regression to investigate associations, and receiver operating characteristic curve analysis to evaluate the predictive accuracy of the body composition measures.

**Results:**

Participants were categorized into frail (n=28), prefrail (n=33), and robust (n=33) groups. All body composition measures exhibited high specificity in detecting frailty, with varying sensitivity. Unadjusted US showed the highest specificity but low sensitivity (10.7%). WBPhA and right leg lean mass (LeanM RL) demonstrated significant predictive capabilities, especially when adjusted for age and sex, with area under the curve values ranging from 0.678 to 0.762. The adjusted LeanM RL model showed a good balance between sensitivity (35.7%) and specificity (93.9%; *P*=.045), indicating its potential as a reliable frailty predictor. These findings are consistent with previous research emphasizing the importance of muscle mass and cellular health in frailty assessment.

**Conclusions:**

Body composition variables, particularly WBPhA, LeanM RL, and US, are effective predictors of frailty in older adults. The PowerFrail mobile app, combined with advanced body composition analysis, offers a practical and noninvasive method for early frailty detection in primary care settings. Integrating such technological tools can enhance the early identification and management of frailty, thereby improving health outcomes in the aging population.

## Introduction

Frailty syndrome in older adults is a multifaceted clinical condition characterized by decreased physiological reserves and increased vulnerability to stressors, which elevates the risk of adverse health outcomes such as falls, hospitalization, disability, and mortality [1,2]. As the global population ages, the prevalence of frailty is projected to rise, posing significant challenges to health care systems worldwide [3]. Early identification and management of frailty are crucial for enhancing quality of life, maintaining functional independence, and reducing health care costs associated with frailty-related complications [4,5].

Primary care facilities are uniquely positioned to play a pivotal role in the early detection and management of frailty due to their accessibility and continuous engagement with the aging population [6]. Implementing effective screening tools within primary care can facilitate timely interventions, thereby mitigating the progression of frailty and its associated adverse outcomes [7]. However, the integration of comprehensive frailty assessments into routine primary care practice remains limited, often due to time constraints, lack of standardized tools, and insufficient training among primary care providers [8].

Body composition variables, particularly those related to muscle mass and tissue quality, are essential in identifying and predicting frailty syndrome [9]. Techniques such as dual-energy X-ray absorptiometry (DXA), bioelectrical impedance analysis (BIA), and muscle ultrasound (US) have been extensively used to assess body composition and muscle status in older adults [10]. The whole-body phase angle (WBPhA), obtained through BIA, serves as an indicator of cellular health and nutritional status, with lower values associated with increased frailty and poorer clinical outcomes [11,12]. Similarly, muscle US offers a noninvasive way to assess muscle thickness and quality, aiding in the detection of sarcopenia and frailty [13]. Although DXA is considered the gold standard for measuring bone and muscle mass, its high cost and limited accessibility in primary care facilities make it necessary to explore alternative assessment methods [14].

In recent years, the advent of mobile health (mHealth) apps has introduced innovative solutions for the assessment and monitoring of complex geriatric syndromes, including frailty. The PowerFrail app represents a pioneering effort in this domain, being the first clinical and scientifically validated app designed to assess muscle power and frailty in older adults in a user-friendly manner [15,16]. This app not only facilitates the screening process but also provides individualized recommendations for improvement and tailored physical activity regimens, thereby supporting personalized intervention strategies. The utilization of validated mobile apps and new trends around artificial intelligence in primary care hold significant promise for enhancing the early detection and management of frailty, offering advantages such as accessibility, ease of use, and the ability to provide real-time feedback and recommendations [17,18].

Moreover, integrating mHealth tools into primary care can bridge gaps in health care delivery by enabling continuous monitoring and follow-up, which are critical for managing chronic conditions and preventing the escalation of frailty [18]. Previous studies have highlighted the effectiveness of mobile apps in improving health outcomes among older adults by facilitating timely interventions and enhancing patient engagement [19]. These findings underscore the potential of mHealth solutions to complement traditional assessment methods, providing a comprehensive approach to frailty management in primary care settings.

From above, the main objective of this study is to analyze the differences in variables related to anthropometric characteristics, physical function, nutritional status, cognitive status, and body composition in phenotypes of frail, prefrail, and robust older adults identified in primary care services. Frailty levels will be classified using the PowerFrail app. Additionally, the second objective is to assess the predictive capacity of body composition variables (US, bioimpedance, and DXA) in identifying older adults with frailty and to evaluate their implementation in primary care services. We used the technological tools WBPhA, US for rectus femoris muscle thickness, and DXA for bone mineral density and lean mass assessment. Our results could demonstrate that these parameters are useful predictors for the identification of frailty, in line with previous findings, thus supporting the potential integration of these tools into primary care practices for the early detection and management of frailty, integrating for the first time mHealth technologies with advanced body composition analysis systems.

## Methods

### Ethical Considerations

This was a descriptive cross-sectional study that evaluated clinical, physiological, body composition, and psychometric variables in a sample of older adult participants. Recruitment and data collection took place between March and June of 2023. Participants were recruited through advertisements from the public Andalusian Health Service system, Spain. Participants were recruited from primary care centers through referrals by health care professionals. Recruitment was conducted in collaboration with general practitioners, who identified potential participants meeting the inclusion criteria. The study adhered to the ethical principles of the Declaration of Helsinki for medical research involving human subjects. All participants provided written informed consent. This study was approved by the Costa del Sol Institutional Ethics Committee, under protocol number BON22, on December 23, 2022. All participants provided written informed consent prior to their inclusion in the study. Participants’ privacy and confidentiality were ensured throughout the study: all data were anonymized prior to analysis, and identifying information was stored securely and separately from the study data. No identifiable images or information of participants are included in this publication. Participants did not receive compensation for their participation..

### Participants

Inclusion criteria for the study were as follows:

- Individuals aged between 70 and 80 years, inclusive.
- Absence or presence of mild cognitive impairment as determined by a Barthel index score [20] greater than 95.
- Sit-to-stand test score [21] between 2.5 and 3.6 for men or between 1.9 and 3 for women.
- Signed informed consent.

Exclusion criteria were the following:

- Individuals younger than 70 years or older than 80 years.
- Individuals with moderate or severe cognitive impairment.
- Sit-to-stand test score [21] less than 2.5 or greater than 3.6 for men or less than 1.9 or greater than 3 for women.
- Individuals living in institutions.
- Individuals with pacemakers or metal prostheses.

### Variables and Procedure

The aim of this study was to identify differences in measured parameters to classify individuals into different states of frailty within primary care. To identify different states of frailty, the sit-to-stand test was used, and relative power was calculated using the equations validated by Losa-Reyna et al [16]. Using the cutoff points determined by Losa-Reyna et al [22], participants were classified into 3 groups: frail, prefrail, and robust. This test was performed in the health center’s office.

After identifying the study subjects, a detailed evaluation of the remaining variables was carried out in the Functional Testing Laboratory of the Physical Education and Sports Area of the University of Málaga. Participants came to the laboratory fasting (a minimum of 3 hours) and without having done any previous exercise, wearing comfortable and light clothing without metal objects. The tests were carried out between 9:30 AM and 2:00 PM. Once in the laboratory, 2 qualified researchers administered the different tests and assessments; body composition determinations were performed fasting and in the early morning, and the rest of the scheduled evaluations were carried out after breakfast.

### Clinical and Demographic Variables

We collected the following clinical and demographic variables:

- Age and sex: Recorded during the initial interview (age in years; sex as male or female).
- Body mass index: Calculated by dividing weight (measured in kilograms using a Digital Scale Extra Large Seca Robusta 813) by height (measured in meters using a stadiometer) squared (m²).
- Number of medications (drugs): Extracted from the clinical history of the Diraya system (Andalusian Health Service database), with the participants’ consent.
- Education level: Recorded during the initial interview.

### Body Composition Variables

We collected the following body composition variables:

- Muscle architecture: Muscle thickness and pennation angle of the rectus femoris muscle of the dominant leg were measured using US (Logiq Book XP Ultrasound System and 8L linear transducer). The rectus femoris was examined with the participant in a supine position, with the US operator standing on the ipsilateral side of the participant. The evaluation protocol previously described by Mateos-Angulo et al [23] was used.
- DXA: Lean mass of arms and legs, total lean body mass, skeletal muscle index, and bone mineral density were determined using a Hologic Horizon A DXA scanner (Hologic Inc). Each subject was examined by a certified technician. The distinction between bone and soft tissue, the detection of edges, and regional demarcations were performed using computer algorithms with APEX Corporation Software (version 5.6.0.7). For each scan, patients were asked to remove all materials that could attenuate the X-ray beam, including jewelry. Due to the sensitivity of the soft tissue analysis, the patient should only wear a paper gown for the scan. There should be no pillow on the scan, as the material would affect the soft tissue measurement. The densitometer calibration was checked daily with the standard calibration block supplied by the manufacturer.
- BIA: Total body water, intracellular, extracellular water, and phase angle (WBPhA) variables were determined by multifrequency bioimpedance using the Inbody 770 model. Multifrequency segmental data were obtained that accurately determined total body water, intracellular and extracellular water, impedance (Xc and R), and phase angle (Z) in the 5 body segments (right arm, left arm, trunk, right leg, and left leg).

### Cognitive and Nutritional Status

To assess cognitive and nutritional status, the following questionnaires were administered digitally using the Google Forms application.

- Cognitive capacity: Cognitive status was evaluated [24] using the General Practitioner Assessment of Cognition, score 0 to 8, a rapid, reliable, and specific test for the detection of dementia in primary care. This instrument is considered an efficient alternative to others, such as the Mini-Mental State Examination, due to its rapid administration and lack of bias related to gender, education level, or mental health.
- Nutritional status screening: Nutritional status was assessed using the Mini Nutritional Assessment (MNA) [25], score 0 to 14, a widely used scale in the geriatric population. The first part of this test serves as a screening tool to detect the risk of malnutrition, with a cutoff value of 10 points or less. It is a widely used method in older adults and has been validated in different clinical contexts.

### Physical Function Evaluation

To evaluate the physical function of the participants, the Short Physical Performance Battery (SPPB) was used, score 0 to 12 [26]. This battery includes three tests:

- Walking speed: Measured over a 4-meter distance, expressed in meters per second. Given that gait speed measurement is used as a tool for detecting frailty in older adults from the general population due to its high sensitivity, simplicity, and feasibility, we also used the cutoff points for frailty and prefrailty related to gait speed and sarcopenia in accordance with the consensus document developed by Cruz-Jentoft et al [27]. In this framework, values below 0.6 m/s are identified as frail, between 0.6 and 1 m/s as prefrail, and above 1 m/s as robust [27].
- Static balance: Assessed in 3 different positions.
- Sit-to-stand test: Evaluation of the time it takes for participants to stand up and sit down from a chair 5 times.

**Figure 1.**
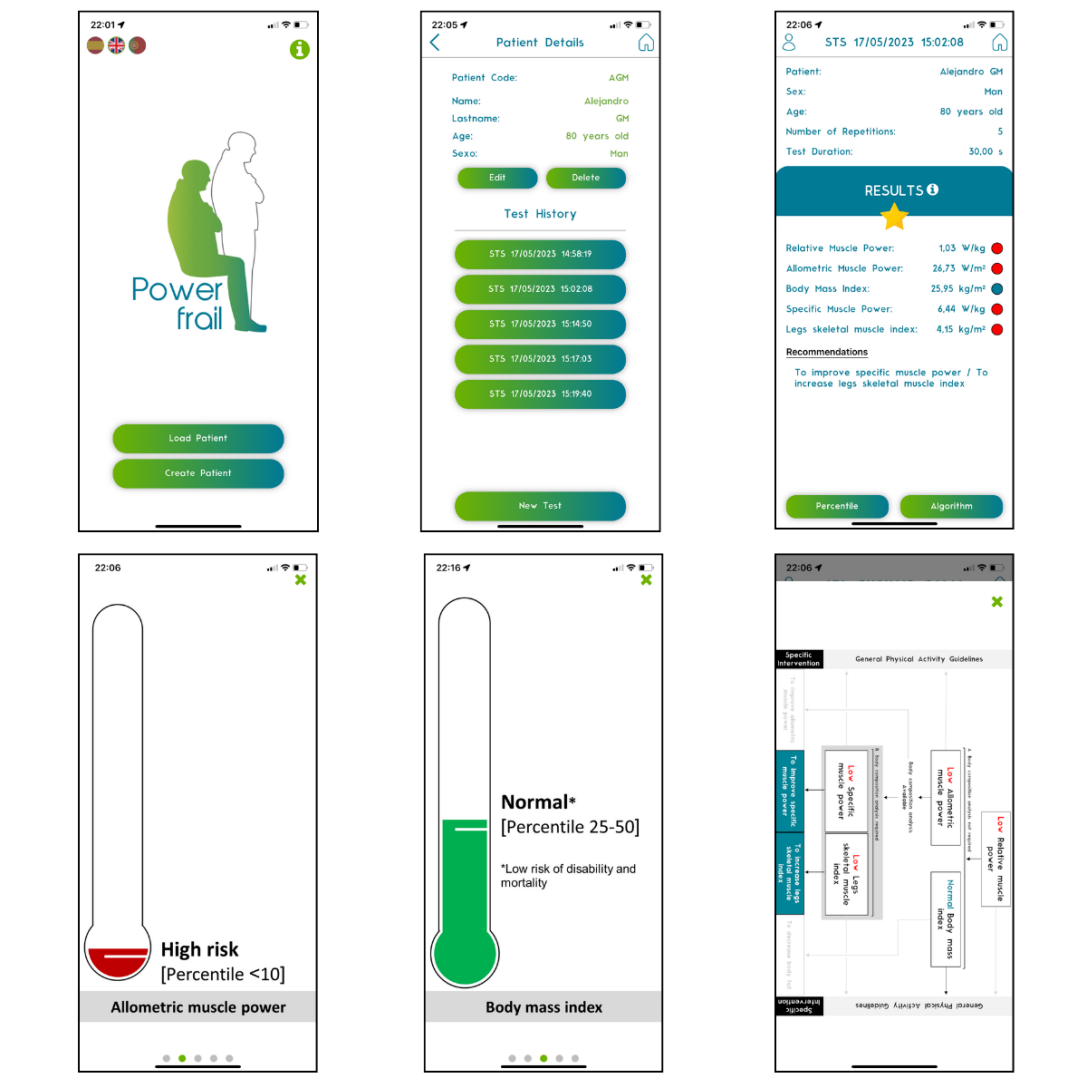
PowerFrail mobile app screenshots.

In addition to the tests included in the SPPB, two tests were performed:

- Mobile lower limb relative muscle power (RPOW): To measure RPOW, participants performed 5 repetitions of standing up from and sitting down onto a chair with a height of 0.46 m, following the protocol validated by Alcazar et al [15]. The test was performed using the PowerFrail app (Figure 1), developed and validated by Losa-Reyna et al [16], installed on a stable smartphone.
- Handgrip strength: Isometric handgrip strength, expressed in kilograms, was measured using a Takei Physical Fitness Test adjustable dynamometer, following a standardized protocol, as indicated by Roberts et al [28]. Participants performed the test in an erect standing position, with shoulders adducted and arms extended parallel to the body, without touching their torso. Two attempts were made for each extremity, and the maximum value was considered, regardless of hand dominance.

### Sample Size Calculation

The sample size was estimated using an ANOVA model for 3 independent groups (robust, prefrail, and frail). The calculation was performed using the G*Power software. For the sample size calculation, a power of 90% (1–β=.90), a significance level of 5% (*α*=.05), and an effect size of 0.4 were used. The result was a total sample size of 84 participants. This size was obtained with a noncentrality parameter (λ) of 13.44 and a critical *F* of 3.11, with 2 degrees of freedom in the numerator and 81 in the denominator. To avoid losses in the initial calculation, enough participants were recruited to maintain the robustness of the study. Finally, 94 subjects participated in the study, distributed across 3 groups: robust, prefrail, and frail.

### Statistical Analysis

To compare the differences on all variables between robust participants and those with frailty and prefrailty, a 1-way ANOVA was performed on the total sample. Logistic regression analysis was performed to investigate the relationship between body composition and frailty. WBPhA, thickness US, and DXA lean muscle from the lower limbs were entered into the regression model as independent variables, as they were found to have significant differences between the studied groups. Considering that age and sex may influence the relationship between body composition and frailty, these factors were introduced as a confounding variable into an adjusted regression model, treating age as a continuous covariate and sex as a categorical factor. The dependent variable was a binary indicator of frailty, coded as 1 for participants with frailty and 0 for robust participants and those with prefrailty.

To extract values for body composition to identify the presence of prefrailty or frailty, we conducted an analysis using the receiver operating characteristic (ROC) curve. This analysis was applied only to body composition that was significant in the logistic regression analysis. In the ROC analysis, the outcome variable was the presence or absence of frailty. The test variable was the body composition that was significantly associated with frailty. Youden index [29] was calculated with the following formula: Youden index = sensitivity + specificity – 1. The area under the curve (AUC), sensitivity, and specificity were calculated to evaluate the accuracy of the identified predictive models. The AUC could distinguish between nonpredictive (AUC<0.5), less predictive (0.5<AUC<0.7), moderately predictive (0.7<AUC<0.9), and highly predictive (0.9<AUC<1) values, as well as perfect prediction (AUC=1) [30].

## Results

The background information of the participants is shown in Table 1. Of the 94 participants in this study, 28 were frail, 33 were prefrail, and 33 had a robust profile. The average age for each frailty category was 76.5, 75.3, and 74.0 years, respectively. The percentages of men and women with frailty and prefrailty were 32% and 52% for men and 68% and 48% for women, respectively. All investigated variables were significantly different among the frailty categories, except BMI, MNA score, skeletal mass index, left arm lean mass, right arm lean mass, and total body lean mass.

**Table 1.** Descriptive characteristics of the sample (n=94).

	Frailty^a^	Prefrailty	Robust	*P* value^b^	Post hoc^a^
Particpants, n (%)	28 (30)	33 (35)	33 (35)		
Sex (female; male), n (%)	19 (68); 9 (32)	16 (48); 17 (52)	17 (52); 16 (48)		
Age (years), mean (SD)	76.46 (2.92)	75.34 (2.86)	73.99 (2.59)	.004	*F*>R
BMI (kg/m^2^), mean (SD)	28.35 (5.34)	27.87 (4.73)	28.65 (5.27)	.82	
RPOW^c^ (W/kg), mean (SD)	1.69 (0.54)	2.60 (0.32)	3.32 (0.46)	<.001	*F*<P<R
MNA^d^ (score, 0‐14), mean (SD)	13.00 (1.28)	12.94 (1.71)	13.30 (0.98)	.52	
GPCOG^e^ (score, 0‐8), mean (SD)	5.46 (1.73)	6.60 (1.32)	7.15 (1.00)	<.001	*F*<P,R
SPPB^f^ (score, 0‐12), mean (SD)	8.07 (2.26)	10.79 (1.24)	11.75 (0.43)	<.001	*F*<P<R
5STS^g^ (s), mean (SD)	18.47 (6.44)	12.01 (1.40)	9.27 (1.31)	<.001	*F*<P<R
WALK4m^h^ (s), mean (SD)	5.92 (2.25)	4.07 (0.75)	3.92 (0.61)	<.001	*F*<P<R
GS4m^i^ (m/s), mean (SD)	0.74 (0.21)	1.01 (0.16)	1.04 (0.15)	<.001	*F*<P,R
Handgrip max (kg), mean (SD)	19.24 (5.39)	25.48 (6.57)	26.25 (7.50)	<.001	*F*<P,R
Number of drugs^j^, mean (SD)	6.89 (2.81)	4.73 (3.13)	3.09 (2.20)	<.001	*F*>P>R
US^k^ (cm), mean (SD)	0.92 (0.32)	1.02 (0.25)	1.16 (0.26)	.004	*F*<R
WBPhA^l^ (°), mean (SD)	4.36 (0.55)	4.63 (0.61)	4.95 (0.43)	<.001	*F*<R
SMI^m^, mean (SD)	6.51 (1.04)	7.09 (1.08)	7.20 (1.33)	.07	
Left arm lean mass (g), mean (SD)	2088.24 (624.67)	2485.68 (720.65)	2408.92 (772.28)	.08	
Right arm lean mass (g), mean (SD)	2274.00 (597.82)	2624.04 (787.75)	2661.02 (915.64)	.12	
Left leg lean mass (g), mean (SD)	5975.11 (1494.27)	7147.72 (1794.43)	7158.07 (2192.25)	.02	*F*<P,R
Right leg lean mass (g), mean (SD)	5982.38 (1573.34)	7368.42 (1823.40)	7314.78 (2040.34)	.006	*F*<P,R
Total body lean mass (g), mean (SD)	42,130.41 (8174.96)	48,123.28 (11,063.12)	47,858.20 (12,130.27)	.06	

a F: frailty; P: prefrailty; R: robust.

b *P* value adjusted for comparing a family of 3.

c RPOW: relative muscle power.

d MNA: Mini Nutritional Assessment.

e GPCOG: General Practitioner Assessment of Cognition.

f SPPB: Short Physical Performance Battery.

g 5STS: 5 sit-to-stand time.

h WALK4m: time to walk 4 meters.

i GS4m: gait speed over 4 meters at a normal pace.

j Drugs: number of daily medications.

k US: ultrasound on rectus femoris.

l WBPhA: whole-body phase angle.

m SMI: skeletal muscle index.

**Figure 2.**
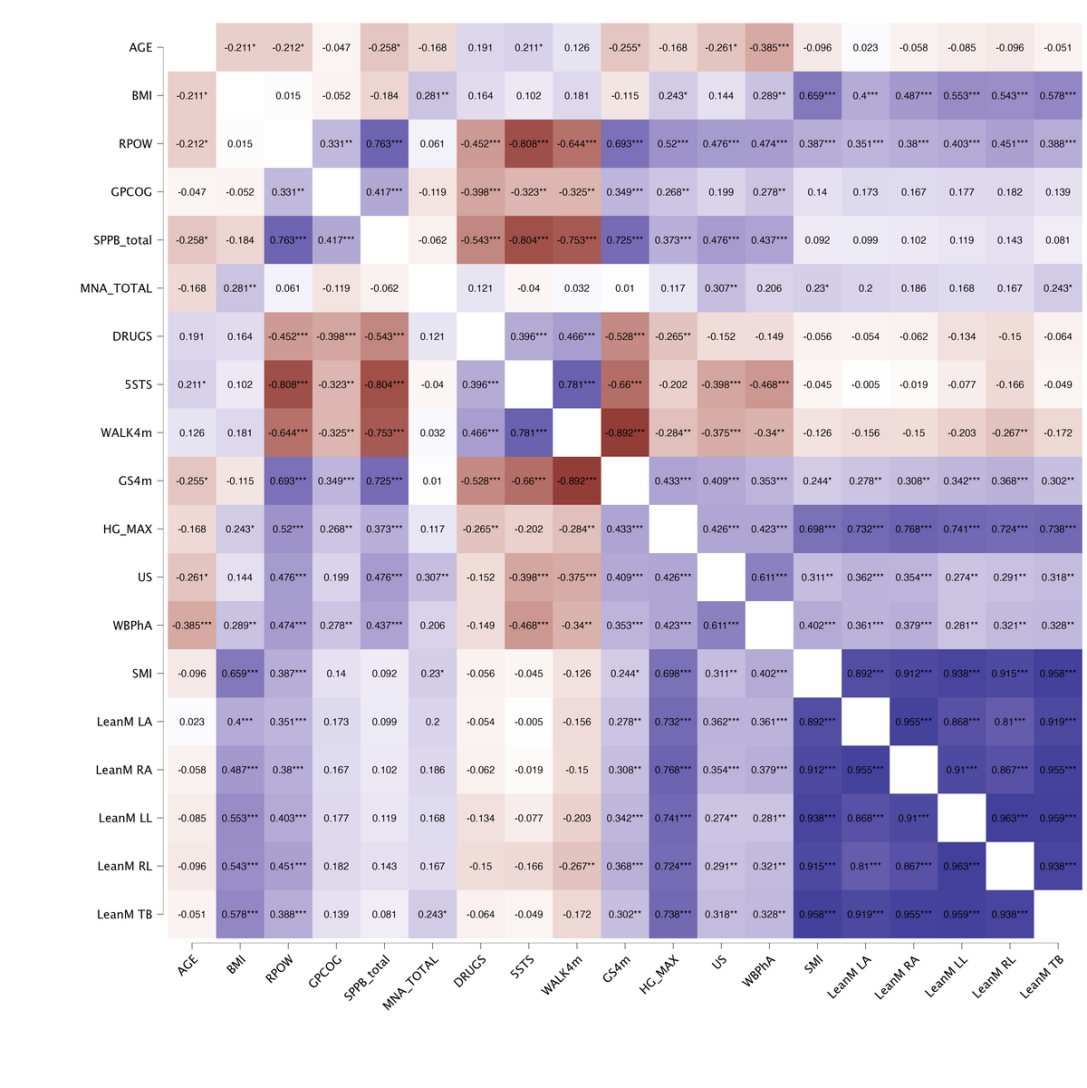
Simple correlation analysis results for the whole sample (n=94). Pearson correlations of all studied variable were carried out on the total sample. 5STS: 5 sit-to-stand time; DRUGS: number of daily medications; GPCOG: General Practitioner Assessment of Cognition score; GS4m: gait speed over 4 meters at a normal pace; HG: handgrip; LeanM LA: left arm lean mass; LeanM LL: left leg lean mass; LeanM RA: right arm lean mass; LeanM RL: right leg lean mass; LeanM TB: total body lean mass; MNA: Mini Nutritional Assessment score; RPOW: relative muscle power; SMI: skeletal muscle index; SPPB: Short Physical Performance Battery score; US: ultrasound on rectus femoris; WALK4m: time to walk 4 meters; WBPhA: whole-body phase angle. *P<.05, **P<.01, ***P<.001.

**Figure 3.**
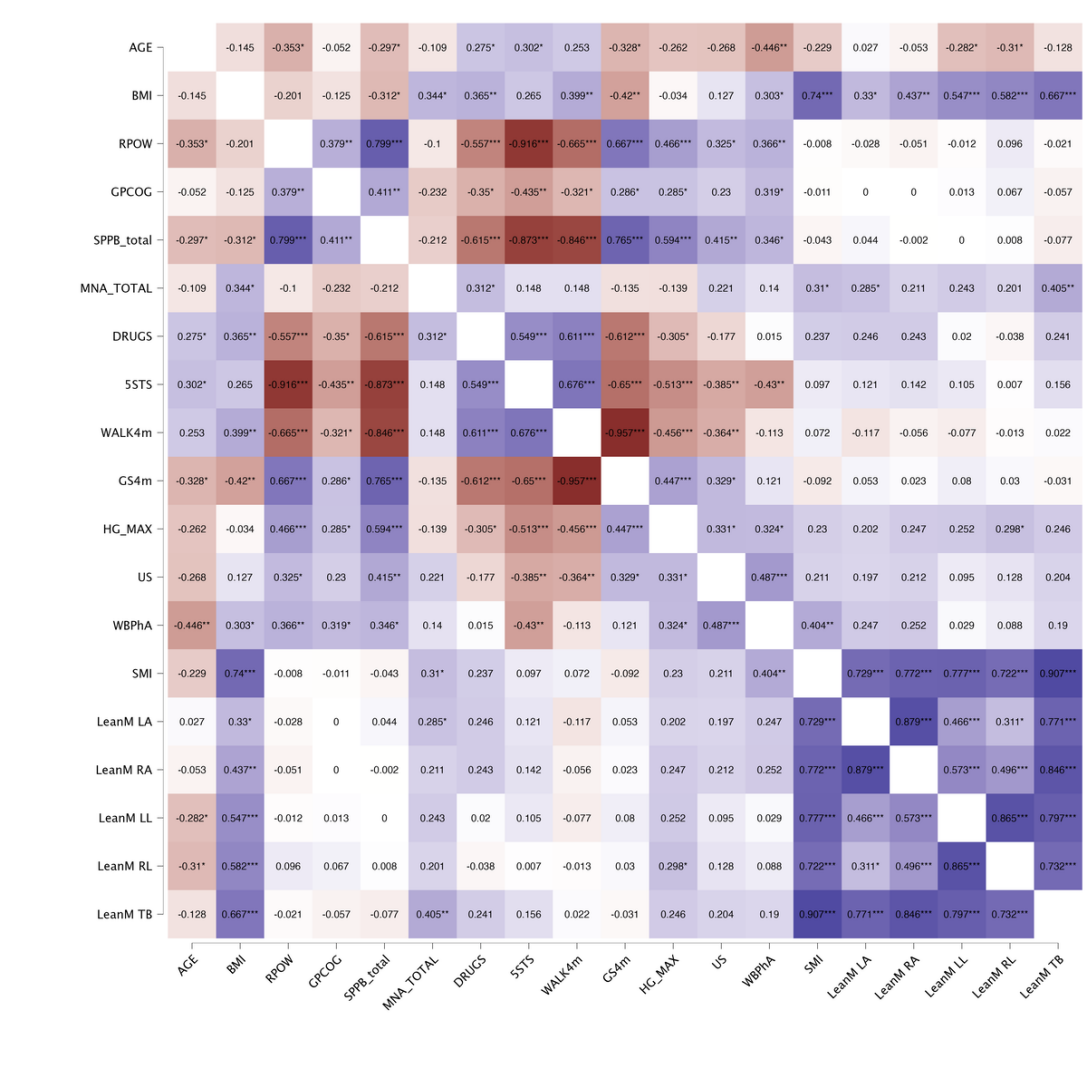
Simple correlation analysis results for women (n=52). Pearson correlations were conducted for all studied variables on the sample of women. 5STS: 5 sit-to-stand time; DRUGS: number of daily medications; GPCOG: General Practitioner Assessment of Cognition score; GS4m: gait speed over 4 meters at a normal pace; HG: handgrip; LeanM LA: left arm lean mass; LeanM LL: left leg lean mass; LeanM RA: right arm lean mass; LeanM RL: right leg lean mass; LeanM TB: total body lean mass; MNA: Mini Nutritional Assessment score; RPOW: relative muscle power; SMI: skeletal muscle index; SPPB: Short Physical Performance Battery score; US: ultrasound on rectus femoris; WALK4m: time to walk 4 meters; WBPhA: whole-body phase angle. *P<.05, **P<.01, ***P<.001.

**Figure 4.**
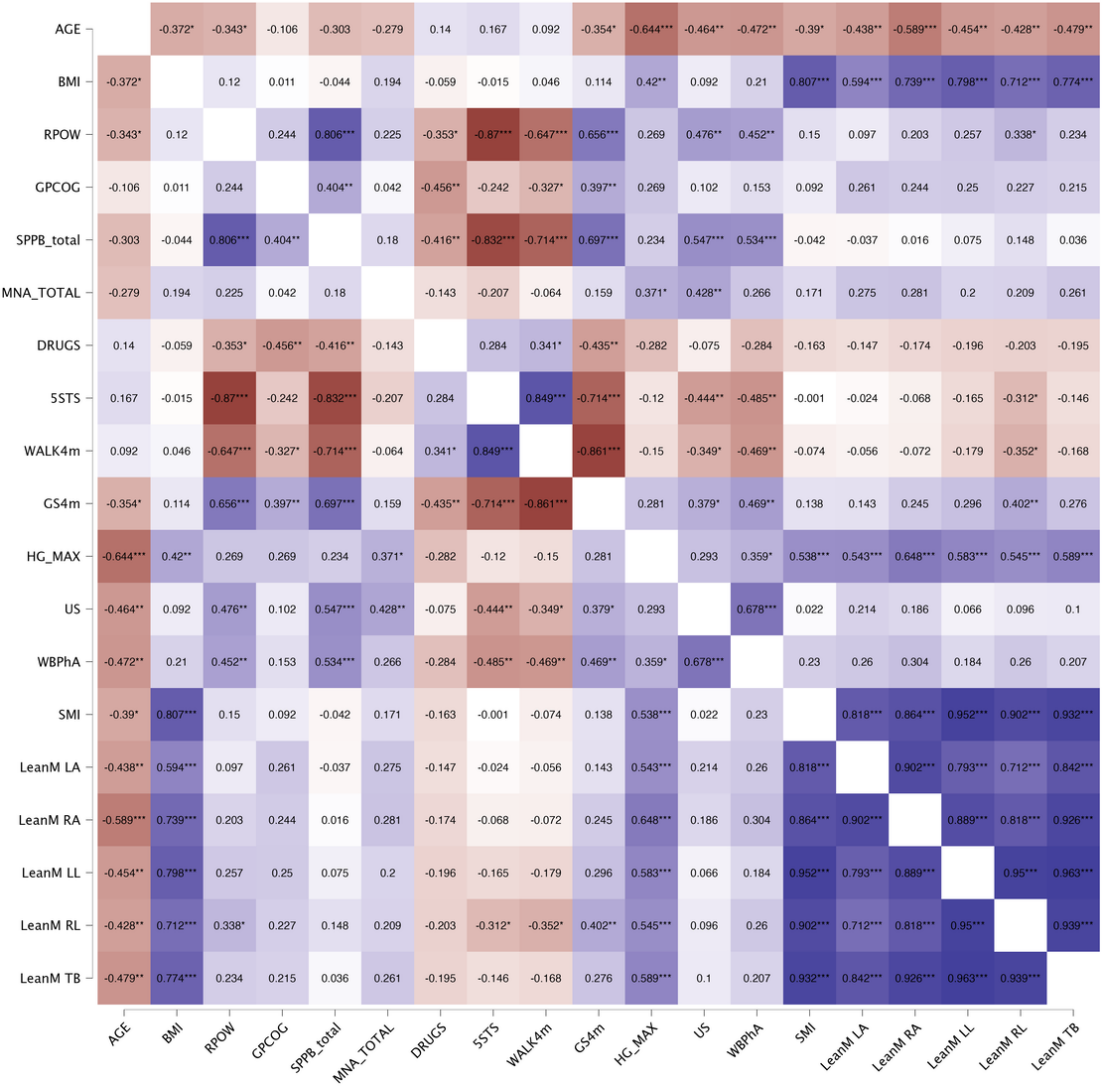
Simple correlation analysis results for men (n=42). Pearson correlations were conducted for all studied variables on the sample of men (n=42). 5STS: 5 sit-to-stand time; DRUGS: number of daily medications; GPCOG: General Practitioner Assessment of Cognition score; GS4m: gait speed over 4 meters at a normal pace; HG: handgrip; LeanM LA: left arm lean mass; LeanM LL: left leg lean mass; LeanM RA: right arm lean mass; LeanM RL: right leg lean mass; LeanM TB: total body lean mass; MNA: Mini Nutritional Assessment score; RPOW: relative muscle power; SMI: skeletal muscle index; SPPB: Short Physical Performance Battery score; US: ultrasound on rectus femoris; WALK4m: time to walk 4 meters; WBPhA: whole-body phase angle. *P<.05, **P<.01, ***P<.001.

The correlation analysis is presented through a heat map in Figures 2-4. Starting with the primary variable identified as a frailty criterion (RPOW), we initially analyzed the behavior of this variable in relation to the other studied variables. Additional correlation analyses were also explored, such as examining the behavior and relationship of handgrip strength. In this regard, we conducted a first analysis on the entire sample (Figure 2), then one each for women (Figure 3) and men (Figure 4). In the entire sample (Figure 1), we found strong positive and negative correlations between the RPOW and all the variables, except MNA score, however, these relationships changed for the women-only and men-only samples. In the complete sample (Figure 2), the results identified stronger correlations between SPPB and RPOW, 5 sit-to-stand time and RPOW, drugs and RPOW, GS4m and RPOW, and handgrip and RPOW. Regarding the analysis of the correlations separately for women and men (Figures3  4), the results were as expected. Specifically, as observed in the cited figures, there were relationships involving body composition variables (BIA, DXA, and US) and expressions of strength, such as lower limb power and, more markedly, handgrip strength. The observed differences in correlations between sexes are consistent with expectations, given the complex biological, hormonal, and social interactions that affect males and females differently during the aging process. It is important to consider these factors when interpreting the results and when designing interventions that address frailty and functional decline in older adults effectively and equitably.

The logistic regression analyses presented in Table 2 demonstrate the predictive capacity of various diagnostic factors for frailty in older adults. The Nagelkerke *R*² values indicate the proportion of variance in frailty status explained by each model, both before and after adjusting for age and sex. The initial model for US shows that US measurements of the rectus femoris explained 10.1% of the variance in frailty (Nagelkerke *R*²=0.101; *P*=.009). After adjusting for age and sex, the explained variance increases to 20.6% (adjusted Nagelkerke *R*²=0.206; *P*=.002), indicating a stronger association when these factors are considered. WBPhA exhibited a significant association with frailty, explaining 16.4% of the variance in the unadjusted model (Nagelkerke *R*²=0.164; *P*=.001). The adjusted model further enhanced the explained variance to 24.5% (adjusted Nagelkerke *R*²=0.245; *P*<.001), underscoring its robustness as a predictive factor. Both left leg lean mass and right leg lean mass (LeanM RL) were significant predictors of frailty. The unadjusted models explained 12.4% (*P*=.003) and 16.4% (*P*<.001) of the variance, respectively. After adjustment, the explained variance increased to 21.5% for the left leg (*P*=.001) and 24.6% for the right leg (*P*<.001).

**Table 2.** Results of the logistic regression analyses (n=94).

	Nagelkerke *R*^2^	*P* value	Adjusted Nagelkerke *R*^2a^	*P* value
Ultrasound on rectus femoris	0.101	.009	0.206	.002
WBPhA^b^	0.164	.001	0.245	<.001
Lean mass, left leg	0.124	.003	0.215	.001
Lean mass, right leg	0.164	<.001	0.246	<.001

a Adjusted Nagelkerke *R*2 included age as a covariable and sex as a factor.

b WBPhA: whole-body phase angle.

The ROC analyses indicated that all evaluated measures demonstrated a high degree of specificity in detecting frailty in older adults. It is notable that unadjusted muscle US demonstrated a high level of specificity, although sensitivity was relatively low. The WBPhA and LeanM RL also exhibited significant predictive capabilities, with AUC and *P* values indicating good discrimination. When adjusting for sex and age, there was a notable improvement in sensitivity, particularly in the case of LeanM RL, which maintained statistical significance (*P*=.045). These findings suggest that the lean mass of the right leg, adjusted for demographic factors, may be a promising indicator for predicting frailty in this population. On the other hand, it is worth noting that the adjusted WBPhA presented the highest Youden index value (0.417), which suggests that it may be the most effective measure in terms of balancing sensitivity and specificity for predicting frailty. Similarly, the adjusted LeanM RL also had a high Youden index (0.296), which establishes it as another promising measure.

## Discussion

### Principal Findings

This study provides significant evidence on the predictive capacity of body composition variables and their relationship with frailty syndrome in older adults. Technology tools such as BIA, WBPhA, US of the anterior rectus quadriceps muscle thickness, and DXA, were used in this study. The older adult frailty status was calculated using the PowerFrail mobile app [15] (Figure 1). This app is the first scientifically based app that allows the assessment of the muscle power and frailty of older adults in a simple way. Our results showed that these parameters are useful predictors for identifying frailty, in line with previous findings.

This study found several significant correlations between variables related to body composition, physical performance, and cognitive status in female participants, but these were not the same as the values observed in their male counterparts. This may be because women experience more functional limitations than men during the aging process [31]. Women tend to suffer a greater decline in physical function with age, which has been attributed to hormonal changes, particularly the decrease in estrogen levels during menopause [32]. Menopause is associated with changes in body composition characterized by an increase in body fat and a progressive decrease in muscle mass and strength [33]. These alterations can lead to a higher prevalence of sarcopenia and frailty in older women compared to men.

Based on these postmenopausal changes, muscle and fat composition might be more closely related to physical performance in women, which we observed in our correlation results from Figures 2-4. Specifically, the associations between body composition measures (evaluated through BIA, DXA, and US) and expressions of strength, such as lower limb power from the mobile app and handgrip strength, were more pronounced in women. This suggests that changes in muscle quality and quantity may have a greater impact on the physical function of older women.

Although previous research has indicated that muscle quality contributes to physical capacity in older adults, the effect of sex differences on this association has not been thoroughly investigated [34]. Some studies have suggested that muscle quality independently predicts physical function in older men but not in women [35]. However, our findings differ, as we observed significant correlations in women. Unlike previous studies, we included analyses of physical function status, which may explain the different results. The underlying mechanisms of these sex-specific differences still need to be investigated and clarified.

Contributing factors may include differences in muscle structure composition, hormonal influences, and neuromuscular activation patterns between men and women [36]. Additionally, the accuracy and sensitivity of measurement techniques, such as BIA and US, may vary between sexes due to differences in body fat distribution and hydration status [37]. Further research is needed to explore these factors and understand how they influence the relationship between body composition and physical function in older adults.

The connection between body composition and frailty indicators has been the focus of extensive research. However, to our knowledge, no studies have simultaneously compared DXA, BIA, and US, specifically for the identification of frailty syndrome in a primary care setting. Our study addresses this gap by evaluating the predictive capacities of these 3 modalities, offering a comprehensive analysis that can inform clinical practice regarding the most suitable and practical tools for frailty assessment in older adults. Although previous studies have compared two modalities, our inclusion of a third (US) provides a broader perspective on body composition assessment tools available for primary care settings. For instance, research has demonstrated that muscle US is an emerging tool for diagnosing sarcopenia, with studies summarizing its diagnostic accuracy [38]. Additionally, studies have evaluated the reliability and validity of sarcopenia diagnosis using BIA compared with the gold standard, DXA, assessing the predictive accuracy of BIA for diagnosis [39,40]. However, these studies did not include US in their comparisons. Rossini-Venturini et al [41] highlighted that the anthropometric prediction equations developed in their study provide a reliable, practical, and low-cost instrument to assess the components that change the most during the aging process, corroborating our findings. This perspective emphasizes the significance of considering diverse elements of body composition in the evaluation of health among older adults. Although some studies have recognized muscle mass, assessed via DXA or BIA, as a critical element in forecasting frailty [42,43], our analysis demonstrated that lower extremity lean mass (left left lean mass and LeanM RL) did reveal a direct relationship in the models. The present results are in line with findings that indicate muscle mass reduction alone does not suffice to predict frailty without factoring in physical performance [44]. In fact, physical performance, which can be measured through functional tests such as gait speed or grip strength, is vital for evaluating frailty status among older adults [45].

There has been a growing acknowledgment in recent literature regarding the significance of assessing both the quantity and quality of muscle. Xu et al [46] demonstrated that body composition, encompassing both muscle mass and quality, is significantly associated with frailty in older adult inpatients. The current literature suggests that it is not enough to analyze the quantity of muscle mass in absolute terms; quality is equally important. Quality can be assessed through methods such as US and BIA, which provide insights into muscle integrity and performance in terms of its functional capacity. In this context, analyzing muscle quality through US could effectively complement DXA, which has traditionally been considered the gold standard in the assessment of body composition. Although DXA provides valuable information on lean mass and fat mass, it does not provide details on muscle distribution and quality, critical for understanding frailty in older adults [47].

Moreover, our findings indicated that muscle strength and gait speed are important indicators of frailty, corroborating the work of Tsukasaki et al [48], who found a strong association between muscle strength, gait speed, and cross-sectional muscle area determined by midthigh computed tomography. These findings reinforce the idea that comprehensive evaluations of muscle function should be integrated into frailty assessments. Therefore, muscle composition emerges as a potential public health assessment by enabling the clinical quantification of muscle mass and an estimation of physical function in the older adult population [49].

Our findings demonstrated that all measures exhibited high specificity but varying sensitivity in detecting frailty (Table 3). Our ROC analysis showed moderate predictive ability for WBPhA, US thickness, and lean mass of legs from DXA, with an AUC between 0.678 and 0.749. This aligns with the results from previous studies [50,51], which emphasized that models combining body composition measurements with physical performance tests can improve the predictive ability of frailty. Unadjusted US showed perfect specificity but low sensitivity (10.7%), indicating it is highly effective at correctly identifying nonfrail individuals but less capable of detecting those who are frail. This aligns with previous studies suggesting that muscle US, while precise in measuring muscle thickness, may have limitations in sensitivity due to operator dependency and variability [52].

**Table 3.** Results of receiver operating characteristic analysis (n=94)

	Sensitivity	Specificity	Youden index^a^	AUC^b^	*P* value
US^c^	0.107	1.000	0.107	0.678	.02
US (adjusted)^d^	0.321	0.939	0.260	0.732	.24
WBPhA^e^	0.240	0.952	0.192	0.704	.003
WBPhA (adjusted)^d^	0.480	0.937	0.417	0.762	.09
LeanM LL^f^	0.107	0.985	0.092	0.683	.009
LeanM LL (adjusted)^d^	0.250	0.924	0.174	0.741	.17
LeanM RL^g^	0.286	0.939	0.225	0.703	.003
LeanM RL (adjusted)^d^	0.357	0.939	0.296	0.749	.045

a Youden index = sensitivity + specificity – 1.

b AUC: area under the curve.

c US: ultrasound on rectus femoris.

d Model adjusted by sex and age.

e WBPhA: whole-body phase angle.

f LeanM LL: left leg lean mass.

g LeanM RL: right leg lean mass.

Adjusting the US measure for age and sex increased sensitivity to 32.1% but reduced specificity to 93.9%, and the adjusted model did not reach statistical significance (*P*=.24). This suggests that while adjustments improve sensitivity, they may not be sufficient to make US a standalone diagnostic tool for frailty in primary care settings. The WBPhA showed an unadjusted sensitivity of 24% and specificity of 95.2%, with an AUC of 0.704 (*P*=.003). After adjusting for age and sex, sensitivity improved to 48%, specificity slightly decreased to 93.7%, and AUC increased to 0.762, although the *P* value was nonsignificant (*P*=.09). These results suggest that WBPhA, particularly when adjusted for sex and age factors, has potential as a screening tool for frailty. Previous research has shown that lower phase angle values are associated with decreased muscle function, poor nutritional status, and higher frailty risk [12,53]. The noninvasive nature and ease of use of BIA make WBPhA a practical option for primary care, although standardization of measurement protocols is necessary.

LeanM RL emerged as a significant predictor when adjusted for age and sex (*P*=.045), with sensitivity and specificity of 35.7% and 93.9%, respectively, and an AUC of 0.749. This indicates that the adjusted LeanM RL model had a good balance between sensitivity and specificity and may be valuable in predicting frailty. The importance of lower limb muscle mass in frailty assessment is well-documented. For instance, [54] reported that decreased appendicular lean mass is associated with physical disability and increased risk of adverse outcomes in older adults. However, the use of DXA in primary care is limited due to cost, accessibility, and exposure to low-dose radiation.

In comparison, the unadjusted left leg lean mass showed similar specificity (98.5%) but low sensitivity (10.7%), and the adjusted model did not achieve statistical significance. This suggests that while lean mass measurements are informative, the right leg may provide more predictive value than the left in this context, possibly due to dominance or functional differences, although further research is needed to confirm this observation. Overall, our results indicated that LeanM RL, adjusted for age and sex, may be the most effective measure among those studied for predicting frailty. This is significant because identifying reliable, accessible markers for frailty is crucial for early intervention. Given the limitations of DXA in primary care, exploring alternative methods to estimate lean mass, such as predictive equations or portable devices, could enhance feasibility. The high specificity observed across all measures suggests they are effective in ruling out frailty in individuals without frailty. However, the variable sensitivity underscores the need for multicomponent assessment tools. The Comprehensive Geriatric Assessment remains the gold standard for frailty evaluation but is resource-intensive [55]. Incorporating measures like WBPhA and simplified lean mass assessments could enhance screening efficiency in primary care.

### Limitations, Clinical Implications, and Future Directions

Limitations of the study include the sample size and homogeneity in some variables, such as BMI and arm lean mass (left arm and right arm), which could have affected the accuracy of the predictive results. Heterogeneity in these measurements has been reported in previous research [56], suggesting that variability in body composition could influence frailty prediction. There is potential for the integration of advanced body composition analysis tools and mobile technology into primary care services for the early identification and management of frailty syndrome in older adults. Methods such as WBPhA, US of the rectus femoris muscle, and DXA analysis proved to be effective predictors of frailty, offering high specificity in detection. The implementation of the PowerFrail mobile app enables rapid and personalized assessments, optimizing preventive interventions. These tools not only improve the accuracy of clinical evaluations but also reduce costs associated with frailty-related complications, promoting healthier aging and alleviating the burden on public health care systems. In addition, while this study incorporated a range of assessment tools, it is essential for future research to concentrate on validating these measures in larger and more diverse cohorts to ensure their wider applicability. The cross-sectional design of this study further restricts the ability to infer causal relationships between body composition variables and frailty. Longitudinal investigations could provide valuable information on how variations in body composition affect the progression of frailty over time. Understanding these dynamics could facilitate developing more effective and personalized interventions for the older adult population. Finally, future studies should explore the integration of these mHealth technologies with advanced body composition analysis systems to optimize early detection and management of frailty.

### Conclusions

The findings of this study reinforce the utility of various body composition evaluations, such as WBPhA, LeanM RL measured by DXA, and quadriceps thickness assessed by US, as effective indicators for predicting frailty in older adults, aligning with previous research. However, our results highlight the necessity of not relying exclusively on muscle mass as a predictor of frailty. It is essential to incorporate assessments of muscle function and physical performance into clinical evaluations to enhance the accuracy of identifying individuals susceptible to frailty. The combination of tools such as WBPhA, bioimpedance, and US, along with the PowerFrail app, could provide a more complete and accurate assessment of the health status of older adults, allowing effective preventive interventions to be implemented in primary care services.

## Abbreviations

AUC: area under the curve
BIA: bioelectrical impedance analysis
DXA: dual-energy X-ray absorptiometry
GS4m: gait speed over 4 meters at a normal pace
LeanM RL: right leg lean mass
mHealth: mobile health
MNA: Mini Nutritional Assessment
ROC: receiver operating characteristic
RPOW: relative muscle power
SPPB: Short Physical Performance Battery
US: ultrasound
WBPhA: whole-body phase angle
